# A Risk-Scoring Model for the Prediction of Endometrial Cancer among Symptomatic Postmenopausal Women with Endometrial Thickness > 4 mm

**DOI:** 10.1155/2014/130569

**Published:** 2014-06-03

**Authors:** Luca Giannella, Kabala Mfuta, Tiziano Setti, Lillo Bruno Cerami, Ezio Bergamini, Fausto Boselli

**Affiliations:** ^1^Local Health Authority of Reggio Emilia, Division of Obstetrics and Gynecology, Cesare Magati Hospital, Viale Martiri della Libertà 6, Scandiano, 42019 Reggio Emilia, Italy; ^2^Institute of Obstetrics and Gynecology, Oncology Prevention Unit, University of Modena and Reggio Emilia, Via del Pozzo 71, 41124 Modena, Italy

## Abstract

*Objective*. To develop and test a risk-scoring model for the prediction of endometrial cancer among symptomatic postmenopausal women at risk of intrauterine malignancy. *Methods*. We prospectively studied 624 postmenopausal women with vaginal bleeding and endometrial thickness > 4 mm undergoing diagnostic hysteroscopy. Patient characteristics and endometrial assessment of women with or without endometrial cancer were compared. Then, a risk-scoring model, including the best predictors of endometrial cancer, was tested. Univariate, multivariate, and ROC curve analysis were performed. Finally, a split-sampling internal validation was also performed. *Results*. The best predictors of endometrial cancer were recurrent vaginal bleeding (odds ratio (OR) = 2.96), the presence of hypertension (OR = 2.01) endometrial thickness > 8 mm (OR = 1.31), and age > 65 years (OR = 1.11). These variables were used to create a risk-scoring model (RHEA risk-model) for the prediction of intrauterine malignancy, with an area under the curve of 0.878 (95% CI 0.842 to 0.908; *P* < 0.0001). At the best cut-off value (score ≥ 4), sensitivity and specificity were 87.5% and 80.1%, respectively. *Conclusion*. Among symptomatic postmenopausal women with endometrial thickness > 4 mm, a risk-scoring model including patient characteristics and endometrial thickness showed a moderate diagnostic accuracy in discriminating women with or without endometrial cancer. Based on this model, a decision algorithm was developed for the management of such a population.

## 1. Introduction

It is known that about 90–95% of postmenopausal women with endometrial cancer report a vaginal bleeding experience [1, 2], whereas about 10% of symptomatic postmenopausal women reveal an intrauterine malignancy [3]. So, a postmenopausal vaginal bleeding is a sign that should not be underestimated. In this regard, a good clinical practice provides, as first diagnostic step, a transvaginal ultrasound in order to discriminate a woman at high or low risk of malignancy.

Usually, an endometrial thickness ≤ 4 mm is a cut-off value for which a conservative management should be adopted. Indeed, in the latter case the posttest probability of having an endometrial cancer drops from 10% to 0.8% [4, 5]. Conversely, among symptomatic postmenopausal women with endometrial thickness > 4 mm, there is an increased risk of cancer [6, 7]. In these cases, further examinations are needed and, usually, an endometrial sampling or an outpatient hysteroscopy should be performed. However, approximately 80–90% of these examinations will not reveal a cancer in a population considered at risk of malignancy [8]. This apparent “inappropriateness” is justified by the fact that our goal is to miss the lowest number of women with cancer.

Despite keeping this important objective in mind, one wonders if there are clinical variables that can improve the diagnostic performance of our procedures. Several studies including patient characteristics or sonographic features were performed in order to test their clinical usefulness. Some authors included, as study participants, all postmenopausal women with vaginal bleeding, whereas other authors included only symptomatic postmenopausal women with an endometrial thickness at risk of intrauterine malignancy [9–13]. The majority of these studies showed fair outcomes with an improvement of diagnostic performance in detecting endometrial cancers. However, to date, these models are not yet validated externally, for which endometrial thickness remains the most important feature to be evaluated in these cases. It is likely that endometrial thickness assessment, along with further predictive factors, could provide better results in the prediction of intrauterine malignancy among high-risk women.

In this regard, the aim of the present study was to create and test a risk-scoring model, including endometrial assessment and patient characteristics, among symptomatic postmenopausal women with endometrial thickness > 4 mm, and furthermore, to develop a decision algorithm for the management of such a population.

## 2. Materials and Methods

This prospective observational study included 624 symptomatic postmenopausal women with endometrial thickness > 4 mm undergoing diagnostic hysteroscopy. The present study was performed at Cesare Magati Hospital, Division of Obstetrics and Gynecology, Scandiano, and University Hospital, Institute of Obstetrics and Gynecology, Modena, Italy, from March 2008 to November 2013. Our Institutional Review Board approved this study and each woman gave an informed consent.

Each postmenopausal woman with vaginal bleeding was subjected to transvaginal ultrasound. The latter examination was performed using a 5–9 MHz vaginal transducer and the thickest part of the anteroposterior bilayer endometrial thickness was measured in the sagittal plane. Furthermore, endometrial echogenicity was evaluated and defined according to the IETA terms (uniform or nonuniform) [14].

Based on our Protocol which suggests further evaluations in all cases with an endometrial thickness > 4 mm, we recruited only those women then subjected to diagnostic hysteroscopy. We excluded all symptomatic postmenopausal women with a vaginal bleeding arising from a cervical or vaginal or vulvar disease, as well as all vaginal bleedings due to hormone replacement therapy (HRT). Conversely, all postmenopausal women under HRT with unscheduled vaginal bleeding were included in the study. Postmenopausal status was defined as the absence of menstruation for at least 12 months after the age of 40 years, where any pathological condition of amenorrhea was excluded.

All eligible women, after transvaginal ultrasound, filled out a questionnaire for their medical history including age; age at menarche; age at menopause; time since menopause; body mass index (BMI = weight (kg)/height^2^ (m^2^)); parity; presence of hypertension or diabetes; HRT, anticoagulant, or tamoxifen use; history of breast cancer; recurrent vaginal bleeding or single episode; endometrial thickness; and echogenicity. Based on previous studies, recurrent vaginal bleeding was defined as any bleeding that lasted seven or more days, or two or more separate episodes of vaginal bleeding over the last year [15].

All symptomatic postmenopausal women with endometrial thickness > 4 mm were subjected to diagnostic outpatient hysteroscopy in vaginoscopy with a saline solution as distension medium and narrow instrumental diameters. The latter examination was performed by an experienced hysteroscopist who was blinded to the ultrasound findings. Each woman was subjected to an endometrial sampling which we considered our reference standard. Based on our previous study [16], a Vabra endometrial sampling was performed in women without any intrauterine lesion; a targeted biopsy along with random biopsies of each uterine wall was performed in women with suspected premalignant or malignant lesion; intrauterine lesion resection was performed in women with polyps or myomas; all women with an atypical endometrial hyperplasia (AEH), as well as all women with an intrauterine malignancy, underwent a hysterectomy which represented our reference standard as definitive histological finding.

The Kolmogorov-Smirnov test was used as test for normal distribution. Nonparametric Mann-Whitney test was performed to compare continuous variables with nonnormal distribution. Categorical variables were evaluated by *χ* ^2^ analysis or Fisher's exact test where appropriate. Variables that showed significant differences in univariate analysis (*P* < 0.05) were the candidate predictor variables for the stepwise logistic regression analysis including both forward and backward selections. In order to create a parsimonious model, we used an entrance and exit *P* value of 0.05/0.05. Then, to test the goodness of fit for the logistic regression model, the Hosmer-Lemeshow test was performed considering the fact that a large value of Chi-squared (with small *P* value < 0.05) indicates poor fit.

In order to overcome some limitations of the stepwise method, such as variable selection, uncertainty about the variables, and overfitting, and based on our sample size (624 women), we performed a split-sampling internal validation [17–19]. We divided our cohort into two, trying to maintain the same number of endometrial cancers in the two halves of our sample, and developed the model on one half (training sample) and tested it on the other (validation sample). We evaluated whether the stepwise regression of the training sample produced the same subset of predictors produced by the regression model of the full dataset [19]. Then, we compared the coefficient of determination (*R* ^2^) between the training and validation sample (*R* ^2^ for the 50% training sample—*R* ^2^ for the 50% validation sample). If the shrinkage was 2% (0.02) or less, validation was considered successful [20]. If so, we derived the final prediction model from the full derivation sample [19]. The coefficient of determination of the training and validation sample was obtained by multiple regression analysis.

Receiver operating characteristic (ROC) curve analysis was used to determine the optimal cut-off value of predictive continuous variables associated with endometrial cancer. According to the predictive odds ratio of each variable obtained in the multivariate analysis, a score for each significant predictive factor was assigned. Then, a ROC curve analysis was performed identifying the score as the variable under study. For each score, sensitivity, specificity, positive predictive value (PPV), negative predictive value (NPV), positive likelihood ratio (LR+), and negative likelihood ratio (LR−) were reported. After considering our disease prevalence (all cases of endometrial cancer) as the pretest probability for endometrial cancer, the likelihood ratio was used to calculate the posttest odds from the pretest odds of disease: posttest odds = pretest odds × likelihood ratio. The relation between odds and probability is odds = *P*/(1 − *P*) and *P* = odds/(1 + odds). Using these equations, we could calculate the posttest probability of disease from the pretest probability of disease [21].

Statistical analyses were performed with MedCalc (MedCalc Software, Mariakerke, Belgium). A *P* value of less than 0.05 was considered to be statistically significant.

## 3. Results

We enrolled 648 symptomatic postmenopausal women with endometrial thickness > 4 mm referred to diagnostic hysteroscopy. 24 women were excluded from this prospective study because a cervical canal stenosis made impracticable an outpatient hysteroscopy for intolerable pain. So, 624 participants were included for our statistical analysis.

Histological examination revealed the presence of 157 (25.2%) women with endometrial atrophy, 275 (44.1%) cases of endometrial polyps, 58 (9.3%) women with submucosal myomas, 62 (9.9%) cases of endometrial hyperplasia (15 cases of complex hyperplasia with atypia, 9 cases of simple hyperplasia with atypia, 22 cases of complex hyperplasia without atypia, and 16 cases of simple hyperplasia without atypia), and 72 women (11.5%) with endometrial cancer.

Patient characteristics showed no significant differences with regard to age at menarche, age at menopause, BMI, parity, diabetes, tamoxifen and anticoagulant use, and breast cancer history (Table 1). Conversely, significant differences were present with regard to age (*P* < 0.0001), time since menopause (*P* < 0.0001), HRT use (*P* = 0.0001), recurrent vaginal bleeding (*P* < 0.0001), presence of hypertension (*P* < 0.0001), endometrial echogenicity (*P* < 0.0001), and endometrial thickness (*P* < 0.0001) (Table 1).

The seven variables that showed significant difference in univariate analysis were included in multivariate analysis (age, time since menopause, HRT use, recurrent vaginal bleeding, presence of hypertension, endometrial echogenicity, and endometrial thickness). Then, stepwise logistic regression analysis showed the significant predictive variables associated with endometrial cancer (acronym, RHEA): R for recurrent vaginal bleeding (OR = 2.96, confidence interval 1.32–6.66, *P* = 0.0084); H for the presence of hypertension (OR = 2.01, confidence interval 1.10–4.50, *P* = 0.0273); E for endometrial thickness (OR = 1.31, confidence interval 1.18–1.45, *P* < 0.0001, criterion > 8 mm); and A for age (OR = 1.11, confidence interval 1.07–1.15, *P* < 0.0001, criterion > 65 years) (Table 2). To test the goodness of fit for the logistic regression model, the Hosmer-Lemeshow test was performed and showed a *P* value of 0.218.

A split-sampling internal validation was performed. The same predictors of the full dataset (recurrent vaginal bleeding, age, endometrial thickness, and hypertension) were produced after the stepwise regression of the training sample. Then, a multiple regression analysis was performed to obtain the coefficient of determination (*R*) for the training and validation sample. The shrinkage between training and validation sample (*R* ^2^–*R* ^2^) was 0.017 (≤2%), and validation was considered successful. We based our interpretation on the model that included all cases.

**Figure 1:**
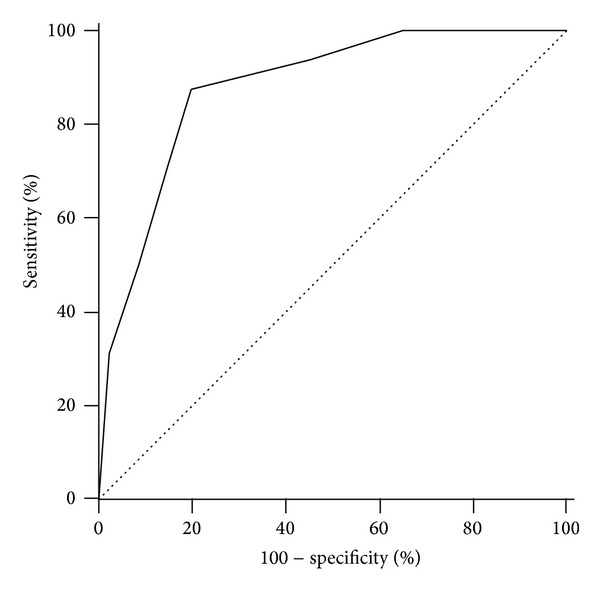
ROC curve associated with the risk-scoring model. The area under the curve was 0.878 (95% CI 0.842 to 0.908; *P* < 0.0001).

According to the predictive odds ratio of each variable obtained in the multivariate analysis, a score for each significant predictive factor was assigned: age > 65 years = 1; recurrent vaginal bleeding = 3; presence of hypertension = 2; endometrial thickness > 8 mm = 1. Then, we built a ROC curve associated with our risk-scoring model. The area under the curve (AUC) was 0.878 (95% confidence interval 0. 842 to 0.908; *P* < 0.0001) (Figure 1). For each score, sensitivity, specificity, PPV, NPV, LR+, and LR− were reported (Table 3). At the best cut-off value (score ≥ 4), sensitivity and specificity were 87.5% and 80.1%, respectively; the PPV and NPV were 36.5% and 98.0%, respectively; LR+ was 4.41 (with a pretest probability of 11.5% and posttest probability of 35.1%); and LR− was 0.16 (with a pretest probability of 11.5% and posttest probability of 1.9%) (Table 3).

## 4. Discussion

According to the accuracy of diagnostic systems [22], the present study showed that a risk-scoring model, including recurrent vaginal bleeding, endometrial thickness > 8 mm, presence of hypertension, and age > 65 years, called RHEA, provided a moderate diagnostic accuracy for the prediction of intrauterine malignancies among symptomatic postmenopausal women at risk of endometrial cancer. At a cut-off score ≥ 4, we obtained a posttest probability of 1.9%, as percentage of missed cancers, and a posttest probability of 35.1%, as percentage of having cancer, from a pretest probability of 11.5%.

Strengths and Weaknesses of the Study. We performed a prospective assessment of our women which allowed us to standardize any type of examination, so as to have more reliable data. Furthermore, all our women had a definitive histological diagnosis with an optimal reference standard. Conversely, it is true that some patient characteristics were collected retrospectively, with clinical questions to our women about past events (e.g., recurrent vaginal bleeding).

We chose symptomatic postmenopausal women with endometrial thickness > 4 mm because women with a lower endometrial thickness have a very low incidence of cancer and, usually, do not perform further examinations in our centers. So, in order not to include in our sample women without a histological diagnosis as reference standard, we selected only women then subjected to hysteroscopy.

In a previous study, including symptomatic postmenopausal women, Bruchim et al. showed that an endometrial thickness of 5–9 mm revealed a cancer in 10% of cases only. For an endometrial thickness > 9 mm, the percentage of cancer reached 18% [23]. These results are in line with the best cut-off value of our prediction model, where an endometrial thickness ≥ 9 mm was one of the predictors associated with endometrial cancer.

In a very interesting study, Opolskiene et al. compared different prediction models for endometrial cancer among postmenopausal women with vaginal bleeding and endometrial thickness ≥ 4.5 mm [24]. They reached the conclusion that, adding endometrial thickness and power Doppler information to patient characteristics, the diagnostic performance of prediction models increased significantly. Concerning this latter study, if we consider only their prediction model including endometrial thickness and clinical variables, we can note that the AUC of their model was similar to that of our risk-scoring model (0.82 and 0.87, resp.). Also Opmeer et al. showed that, taking into account patient characteristics (age, time since menopause, BMI, and diabetes) and endometrial thickness, the appropriateness of their procedures improved significantly. In the latter case, the AUC of their model reached a value of 0.90 [25].

There was a previous study showing a risk-scoring model (Norwich DEFAB) for endometrial cancer including patient characteristics and endometrial thickness [15]. The authors included a very large sample size (3047 postmenopausal women), recruiting all symptomatic postmenopausal women with the assumption that all women with endometrial thickness < 5 mm did not have an intrauterine cancer. Despite the presence of several differences compared to our study, such as studied population, sample size, and disease prevalence, there are many similarities between their results and ours. In this regard, also their best predictor for endometrial cancer was a recurrent vaginal bleeding (OR = 3.93) to which a score of 4 was assigned. The best cut-off value concerning the women's age was similar to ours, with a higher risk of cancer for women over 64 years of age (score = 1). For both models, endometrial thickness was a fair predictor of intrauterine malignancy, but at different cut-off values (≥14 mm versus ≥9 mm, resp.). Conversely, in our univariate analysis diabetes and BMI, which were significant predictive factors for intrauterine malignancy in Burbos's study, there was no statistical difference between women with or without endometrial cancer. Similar results were shown by Opolskiene et al. in their study, where there was no difference in terms of BMI and diabetes in univariate analysis between women with and without cancer [24]. Conversely, as reported by other authors on the same topic [26, 27], our results showed as a good predictor of intrauterine malignancy the presence of hypertension, to which a score of 2 was assigned.

Based on their model and results, Burbos et al. proposed, as discriminatory cut-off point, a score ≥3 which showed a LR+ of 1.64 and LR− of 0.36. Based on this value, the authors proposed a helpful algorithm with several management options for symptomatic postmenopausal women [15].

**Figure 2:**
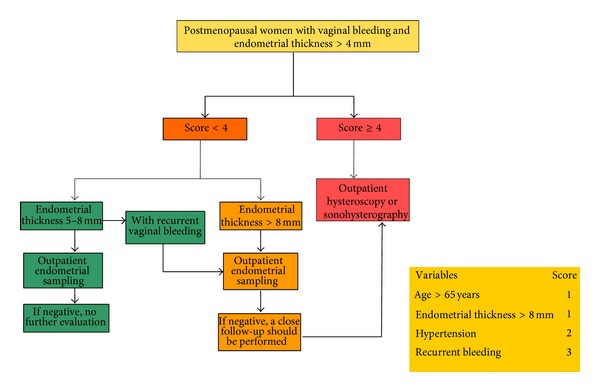
Flow-chart showing a decision algorithm for the management of symptomatic postmenopausal women with endometrial thickness > 4 mm.

Our risk-scoring model, at the best cut-off score (≥4), showed a fair LR− (0.16) with a posttest probability for endometrial cancer of 1.9%. Given the fact that our first objective should be to decrease the number of missed cancer, this score value had a good diagnostic yield for that purpose. A score ≥ 4 means that, at least, there is a woman with an endometrial thickness ≥ 9 mm and recurrent vaginal bleeding. In that case we recommend performing an outpatient hysteroscopy or sonohysterography, because the posttest probability for cancer was 35.1% (LR + 4.41). The latter finding, from a statistical point of view, showed that our model decreased also the false positives, given the pretest probability for endometrial cancer of 11.5%. The issue is much more controversial when the score is less than 4. As mentioned previously, the probability of having cancer was low but present (1.9%) and, according to the algorithm proposed by Burbos et al., we suggested some management options (Figure 2). If endometrial thickness is 5–8 mm, without the presence of recurrent vaginal bleeding (the strongest predictor of endometrial cancer), an outpatient endometrial sampling should be performed and, if negative, no further evaluation should be made. If endometrial thickness is > 8 mm, an outpatient endometrial sampling should be performed and, if negative, a close follow-up with further ultrasound or endometrial sampling could be proposed; an outpatient hysteroscopy or sonohysterography could be also performed. The same management should be adopted for women with endometrial thickness of 5–8 mm and recurrent vaginal bleeding.

This clinical approach makes possible a risk assessment focusing on a more comprehensive clinical evaluation, rather than on endometrial evaluation alone. In this regard, for example, a hypertensive woman of 70 years of age with recurrent vaginal bleeding and endometrial thickness of 4 mm should perform a diagnostic hysteroscopy because, despite her endometrial thickness, she would be more at risk for an intrauterine malignancy.

## 5. Conclusion

Adding some patient characteristics to endometrial thickness, we built a risk-scoring model (RHEA risk-model) with a moderate diagnostic accuracy in detecting intrauterine malignancies among symptomatic postmenopausal women with endometrial thickness > 4 mm.

However, we want to emphasize that, at present, our results are not generalizable and further studies of external validation are mandatory.

## Figures and Tables

**Table 1:** Univariate analysis comparing clinical variables and endometrial assessment between women with (*n* = 72) or without (*n* = 552) endometrial cancer.

Variables	Women with endometrial cancer *n* (%)	Women without endometrial cancer *n* (%)	*P *value
Age (years)*	69 (66–71)	59 (55–65)	<0.0001^a^
Age at menarche (years)*	12 (12-13)	12 (11–13)	0.29^a^
Age at menopause (years)*	52 (50–53)	52 (50–53)	0.86^a^
Time since menopause (years)*	17 (17-18)	7 (4–14)	<0.0001^a^
BMI*	28 (25–31)	28 (27–31)	0.16^a^
Parity			0.22^b^
Nulligravid	12 (16.6)	132 (23.9)	
Parous	60 (83.4)	420 (76.1)	
HRT use			0.0001^b^
Yes	0 (0)	108 (19.5)	
No	72 (100)	444 (80.5)	
Vaginal bleeding			<0.0001^b^
Single episode	24 (33.3)	348 (63.0)	
Recurrent episode	48 (66.7)	204 (37.0)	
Hypertension			<0.0001^b^
Yes	48 (66.7)	208 (37.6)	
No	24 (33.3)	344 (62.4)	
Diabetes			0.88^b^
Yes	12 (16.6)	84 (15.2)	
No	60 (83.4)	468 (84.8)	
Tamoxifen			0.097^c^
Current users	0 (0)	0 (0)	
Past users	0 (0)	24 (4.3)	
Never users	72 (100)	528 (95.7)	
Anticoagulant use			0.53^b^
Yes	18 (25.0)	116 (21.1)	
No	54 (75.0)	436 (78.9)	
Breast cancer			0.097^c^
Yes	0 (0)	24 (4.3)	
No	72 (100)	528 (95.7)	
Endometrial echogenicity			<0.0001^b^
Uniform	0 (0)	200 (36.2)	
Nonuniform	72 (100)	352 (63.8)	
Endometrial thickness (mm)*	11 (9–13)	8 (6–10)	<0.0001^a^

*The values are expressed by median and interquartile range. ^a^Using Mann-Whitney test; ^b^using Chi-square analysis; ^c^using Fisher's exact test; BMI: body mass index; HRT: hormone replacement therapy.

**Table 2:** Multivariate analysis showing clinical and endometrial variables associated with intrauterine malignancy.

Variables	Odds ratio	95% CI	Criterion	*P *value^a^
Age	1.11	1.07–1.15	>65 years	<0.0001
Recurrent vaginal bleeding	2.96	1.32–6.66	—	0.0084
Endometrial thickness	1.31	1.18–1.45	>8 mm	<0.0001
Presence of hypertension	2.01	1.10–4.50	—	0.0273

^a^Using stepwise regression analysis. CI: confidence intervals.

**Table 3:** Sensitivity, specificity, PPV, NPV, LR+, LR−, pre-, and posttest probability for each score of our risk-scoring model.

Cut-off score	Sensitivity (%)	Specificity (%)	PPV (%)	NPV (%)	LR+	LR−	Pretest probability	Posttest probability
≥0	100	0.0	11.5	—	—	—	11.5%	—
≥1	100	21.7	14.3	100	—	0.00	11.5%	0.0%
≥2	100	34.8	16.7	100	—	0.00	11.5%	0.0%
≥3	93.7	54.9	21.3	98.5	—	0.11	11.5%	1.3%
**≥4**	**87.5**	**80.1**	**36.5**	**98.0**	**—**	**0.16**	**11.5%**	**1.9%**
≥5	70.8	85.3	38.6	95.7	—	0.34	11.5%	4.0%
≥6	50.0	91.3	42.9	93.3	—	0.55	11.5%	6.3%
≥7	31.2	97.5	62.5	91.6	—	0.70	11.5%	7.9%
>7	0	100	—	88.5	—	1.00	11.5%	11.5%

≥0	100	0.0	11.5	—	1.00	—	11.5%	11.5%
≥1	100	21.7	14.3	100	1.28	—	11.5%	13.6%
≥2	100	34.8	16.7	100	1.53	—	11.5%	15.8%
≥3	93.7	54.9	21.3	98.5	2.08	—	11.5%	20.3%
**≥4**	**87.5**	**80.1**	**36.5**	**98.0**	**4.41**	**—**	**11.5%**	**35.1%**
≥5	70.8	85.3	38.6	95.7	4.83	—	11.5%	37.2%
≥6	50.0	91.3	42.9	93.3	5.75	—	11.5%	41.4%
≥7	31.2	97.5	62.5	91.6	12.8	—	11.5%	61.1%
>7	0	100	—	88.5	—	—	11.5%	—

PPV = positive predictive value; NPV = negative predictive value; LR+ = positive likelihood ratio; LR− = negative likelihood ratio.
